# Generation of Nanodroplet Reactors and Their Applications in In Situ Controllable Synthesis and Transportation of Ag Nanoparticles

**DOI:** 10.1002/advs.202002672

**Published:** 2021-01-29

**Authors:** Guanhua Lin, Haifei Wang, Wensheng Lu

**Affiliations:** ^1^ Institute for Advanced Study Shenzhen University Nanhai Avenue 3688 Shenzhen Guangdong 518060 China; ^2^ Beijing National Laboratory for Molecular Sciences CAS Key Laboratory of Colloid Interface and Chemical Thermaldynamics Institute of Chemistry Chinese Academy of Sciences Beijing 100190 China

**Keywords:** directed motions, in situ transmission electron microscopy, nanodroplets, nanoreactors, transporting

## Abstract

Nanodroplets have been paid great attention as they are crucial for a wide range of physical, chemical, and biological applications. In this paper, monodispersed nanodroplets are prepared and their directed motions are realized through conducting the formation of nonuniform structures via altering the ionic distribution within; all these dynamics have been observed by using in situ transmission electron microscopy liquid cell technology. It has been found that their transformation from random motion to directed motion is reversible. Moreover, combining multiple directed motions enables long‐distance travel with directed motion taking up 95% of the total time. The results here also prove that aqueous nanodroplets can slide directionally on the hydrophilic surface like droplets sliding on hydrophobic surface. Furthermore, the authors successfully achieve the unidirectional transportation of in situ prepared Ag nanoparticles by using the nanodroplets as nanoreactor, carrier, and transporter. The size and number of as‐prepared Ag nanoparticles can be quantitatively controlled. In summary, this research provides a new strategy for real‐time generation and precise manipulation of aqueous nanodroplets. Together with the quantitatively controllable in situ synthesis of Ag nanoparticles within the nanodroplets, this work can prove their promising applications in many fields.

## Graphical abstract

Schematic figure presents the nanodroplet in the transmission electron microscopy (TEM) liquid cell. In situ TEM images show the coalescence of nanodroplets and their applications in preparing and transporting Ag nanoparticles.

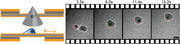

## 1 Introduction

Droplets are ubiquitous in daily life such as rain drops, tears, and blood splats, which also play an important role in many fields as their motion is related to numerous applications including DNA sequencing,^[^ ^1^ ^]^ microchemical reactors,^[^ ^2^ ^]^ printing,^[^ ^3^ ^]^ condensation,^[^ ^4^ ^]^ microfluidics,^[^ ^5^ ^]^ drug delivery,^[^ ^6^ ^]^ and water collection.^[^ ^7^ ^]^ Therefore, studies on droplets have been conducted over the past 50 years in the field of life, health, and physical sciences.^[^ ^8^ ^]^ Among them, directional transportation and/or positioning of liquid droplets on a solid substrate is not only an interesting natural phenomenon, but also a scientific research hotspot,^[^ ^9^ ^]^ especially for the study of transporting nanoscale droplets as their potential applications expands across different fields.^[^ ^10^ ^]^ Recently, interest in fabricating microfluidic chips, micromixers, microactuators, and microfluidic transmissions requires developing smaller and more accurate micro‐/nano‐fluidic systems, which urges a deeper understanding of the formation mechanism and manipulation strategy of the nanoscale droplets.^[^ ^11^ ^]^ Therefore, seeking new strategies for preparing and manipulating nanodroplets under general conditions and environments is necessary. On the other hand, nanoscale materials would present unique properties from their bulk materials. For example, the hydrodynamics of macroscopic droplets is determined solely by visco‐capillary effects, but that of nanodroplets is dominated by disjoining pressure effects.^[^ ^12^ ^]^ Therefore, the macroscopic laws should be validated before applied to nanoscale droplets. Our research aimed at conducting the directed motion of aqueous nanodroplets on the hydrophilic surface will commendably promote the understanding of droplets at nanoscale and lay the foundation for further studies and applications.

Many experimental and theoretical studies have been dedicated to manipulating the droplets through different mechanisms and ways for achieving the directed motion. The most challenging task is to realize effective unidirectional droplet motion. Generally, the directed movements of droplet are induced by unbalanced interfacial tension via introducing a gradient force through external stimuli, such as chemical,^[^ ^13^ ^]^ thermal,^[^ ^14^ ^]^ topographic gradients,^[^ ^15^ ^]^ electric field,^[^ ^16^ ^]^ or their combinations.^[^ ^17^ ^]^ Most of them rely on conducting the directed motion of nanodroplets under specific conditions or hydrophobic environments, such as observing slipping motion of water droplets on superhydrophobic surfaces,^[^ ^18^ ^]^ which limits their practical applications. For instance, when nanodroplets are used in biological systems, it is difficult to exert hydrophobic surfaces, and the applying of thermal gradients would bring unrecoverable damage to the biomolecules.^[^ ^19^ ^]^ Recently, a new strategy has been used for conducting the directed motion of macro droplets. The driving force is generated from the difference of the contact angle between two sides of a droplet, which is realized by altering the distribution or orientation of the solute molecules in the droplets. Various ways can be used to generate difference of their distribution or orientation, such as the spreading change of the molecules,^[^ ^20^ ^]^ and variation of assembled structure of small ionic surfactant molecules.^[^ ^21^ ^]^ These methods display many advantages, including suitability for hydrophilic environment and good reversibility. Therefore, we extend this idea to aqueous nanodroplets and explore a new strategy to achieve long‐distance directed motion under hydrophilic environments, which can further improve our understanding of nanodroplets and achieve their effective unidirectional droplet motion.

Because the size of nanodroplets is too small for optical observation, in situ transmission electron microscopy (TEM) liquid cell technology has been used. This method allows imaging the dynamics of nanoscale substances in liquid phase,^[^ ^22^ ^]^ and it has been confirmed that electron beam did not affect the motion of nanodroplets.^[^ ^23^ ^]^ Herein, we introduce ethylene diamine tetraacetic acid disodium salt (EDTA) aqueous solution for forming nanodroplets and provide a new strategy for triggering their transition from random to directed motions on hydrophilic Si_3_N_4_ surfaces, and in situ visualizing their dynamics by using in situ TEM liquid cells.^[^ ^24^ ^]^ We in situ observed the generation process of nanodroplets, as well as their random and directed motion. Moreover, we successfully achieved the unidirectional and controllable synthesis and transportation of in situ prepared Ag nanoparticles by using these nanodroplets. Meanwhile, our results on the dynamics of splitting and coalescence of nanodroplets can shed new light on how far nanoscale droplets deviates from the macroscopic laws, which is of importance for further studies and applications of nanodroplets. In a word, our research provides a new and simple strategy for preparing and precisely manipulating liquid nanodroplets, and the quantitatively controllable synthesis and transportation of Ag nanoparticles suggests that these nanodroplets can play an important role in many fields.

## 2 Results and Discussion

**Figure 1:**
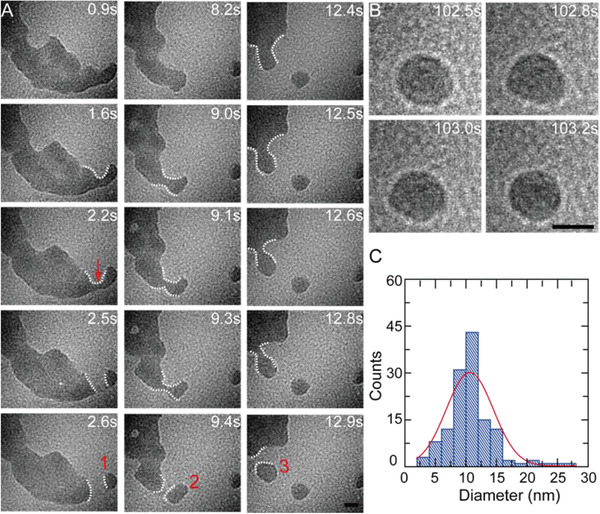
A) In situ TEM images show the dynamic process of three EDTA nanodroplets generated from the liquid layer. B) In situ TEM images show the self‐shaping process of the prepared nanodroplet. C) Histogram of the size of nanodroplets (10 ± 5 nm), measured from ≈130 droplets. The scale bar is 10 nm.

Under continuous exposure to the TEM electron beam, the EDTA solution retracts and forms a thin liquid film on the hydrophilic Si_3_N_4_ membrane. As shown in Movie S1, Supporting Information, the in situ observations show that these nanoscale liquid layers are constantly moving accompanied by fast shape changing owing to their higher surface ratio, and the shape changing presents repeated spread‐withdrawal cycle behavior and each cycle completed within several seconds. This is totally different from liquid macrodroplets which cannot be disturbed easily once they reached a steady state.^[^ ^25^ ^]^ If these aqueous nanolayers are further exposed to intense electron flux (greater than 120 e [Å^2^·s]^−1^), the water would rapidly retract. When the retracting speed is fast enough that partial liquids cannot follow the step, then these liquids can split and generate small nanodroplets. **Figure** **1**A presents in situ TEM images showing the splitting of a nanoscale liquid layer and the formation process of three nanodroplets. It can be seen the liquid layer is stretched beyond a critical elongation magnitude before splitting due to the high specific surface energy. When the capillary forces are unable to restore its original state, the capillary bridge (red arrow in Figure 1A) shrink and eventually break, and then the splitting process is completed leading to the formation of a new nanodroplet. Our observation is in accordance with the splitting process of macroscopic liquid droplets.^[^ ^26^ ^]^

Generally, the initial shapes of the generated nanodroplets were irregular, but these nanodroplets can reshape themselves into a circular shape gradually, even though they were not perfect circles. And the transformation process from an irregular shape to a circular shape was generally completed within seconds. Take nanodroplet 2 as an example, it was elongated right after detaching from the liquid layer (*t* = 9.4 s), then it changed to circular shape within 2 s (*t* = 11.1 s, see Movie S1, Supporting Information). But the circularity of nanodroplets continued to fluctuate owing to the high specific surface energy associated with their movements, as shown in Figure 1B. The change of surface energies (∆*E*) before and after splitting process could be calculated by:

(1) $$\Delta E=\gamma \left(\Delta A_{L}+A_{1}+A_{2}+A_{3}\right)$$

where *γ* is the surface tension, Δ*A*_L_ is the area change of liquid layer, *A*_1_, *A*_2_, and *A*_3_ are the areas of the new nanodroplets. We can measure the area of liquid layer and nanodroplets, giving ∆*A*_L_ = 2322 − 2545 = −223 nm^2^, *A*_1_ = 80 nm^2^, *A*_2_ = 106 nm^2^, and *A*_3_ = 137 nm^2^. Together with *γ* = 0.0728 N m^−1^, we calculate ∆*E* = 7.28 × 10^−18^ J. In other words, the average surface energy for splitting a nanodroplet from liquid layer is ≈2.4 × 10^−18^ J. Histogram of the diameter of these nanodroplets shows that most of the nanodroplets have a diameter of ≈5–15 nm, and the smallest one has a diameter of ≈3 nm during our observation. The average diameter is ≈10 nm, as shown in Figure 1C. It has been found that the smaller nanodroplets (less than ≈10 nm) possess better circle shape, as shown in Figure S1, Supporting Information. Interestingly, we have observed hollow nanodroplets with diameter of ≈27 nm, containing ≈10 nm‐in‐diameter “gas core” inside. Though we are not yet clear about forming mechanism and dynamical process of these hollow nanodroplets, it does not hinder their value as they have excellent potential applications in many critical fields.^[^ ^27^ ^]^

**Figure 2:**
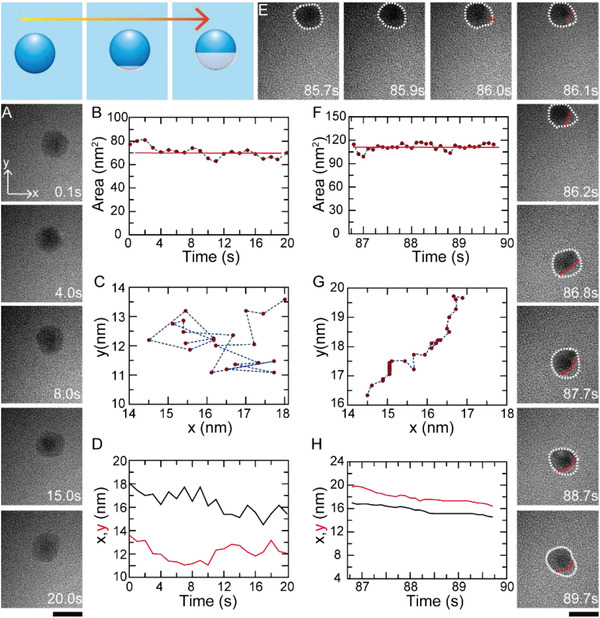
A) In situ TEM images show the random movement of EDTA nanodroplets along with its corresponding B) surface area, and C,D) paths during 20 s. E) In situ TEM images present the transformation process from random motion to the directed motion of the same nanodroplets (A–D) along with its corresponding F) surface area and G,H) paths. The scale bar is 10 nm.

**Figure** **2**A shows a typical real‐time movement (20 s) of ≈10 nm‐in‐diameter nanodroplet on the Si_3_N_4_ surface. The fluctuation of surface area around ≈70 nm^2^ suggested that the self‐shaping process is accompanied with spread‐withdrawal behavior, as shown in Figure 2B. By observing the trajectory of the droplet, it was found that the motion was not confined to a direction but followed a random path (Figure 2C). Another trajectory for the droplet random motion was analyzed and depicted in Figure 2D. All trajectories were found to be different, and the nanodroplet moved in random directions. The random motion, which resembles the 2D Brownian motion, can be attributed to the combined effects of the hysteresis‐free surface and the Marangoni stress. The former was provided by the total wetting property of the substrate and the latter was resulted from the nonuniform distribution around the droplet periphery. Owing to this compensation mechanism, the surface tension at the border fluctuates around a mean value, and its nonuniformity leads to random motion of the droplet.^[^ ^28^ ^]^ The time versus the velocity of the nanodroplet indicated the speed of the random motion could be varied about tenfold. However, it is impossible to exactly predict the time *t* and position (*x*, *y*) of stochastic dynamics of random motion at the same time according to the inertial Langevin law. So, triggering the directed motion to take advantage of them in transporting nanoscale substances is necessary.

In order to trigger the directed motion of these nanodroplets on hydrophilic solid surface, we irradiated the nanodroplet by the same electron beam of the TEM and continuously imaged their movements, as shown in Figure 2E–H. There is a turning point at *t* = 86.0 s, before which nanodroplet still carries on random motion. After *t* = 86.0 s, the nanodroplet exhibits small fluctuations in mass distribution under a uniform electron beam during its self‐shaping process and eventually forms a nonuniform structure. The resulting mass distribution allows us to identify two distinguishable (heavy mass and light mass) parts in the nanodroplet; the nanodroplet then slide toward the heavy mass part direction. In order to better image the dynamics of the nanodroplet, we moved the place and let the nanodroplet on the center. As shown in Figure 2F, the surface area of the nanodroplet fluctuated around ≈110 nm^2^, ≈40 nm^2^ larger than before, which proved the spreading of EDTA nanodroplet on the Si_3_N_4_ surface. The trajectory of the droplet from *t* = 86.8 s to *t* = 89.7 s (Figure 2G,H) indicates a directed motion distinct from the random motion shown in Figure 2C,D. We confirmed these two motions by analyzing the mean square displacement (MSD) of the nanodroplets during each stage. For the trajectory shown in Figure 2A, MSD presents liner relationship with time at the initial time as shown in Figure S2A, Supporting Information, confirming that the nanodroplet experienced random motion. And the diffusion coefficient is ≈1.2 × 10^−19^ m^2^ s^−1^. However, at the movement stage shown in Figure 2E, MSD presents parabola relationship with time as shown in Figure S2B, Supporting Information, which proved that the droplet is in the state of directed motion.^[^ ^29^ ^]^ And the velocity of directional motion is calculated to be ≈1.2 nm s^−1^. In a word, even though the directed motion was recorded at a very short time (just 2.9 s) and narrow range (*x*, *y* ≈ 4 nm), our experimental results first confirmed that nanoscale aqueous droplets can continuously slide on hydrophilic surfaces, just like their sliding movements on hydrophobic surfaces.^[^ ^30^ ^]^

**Scheme 1:**
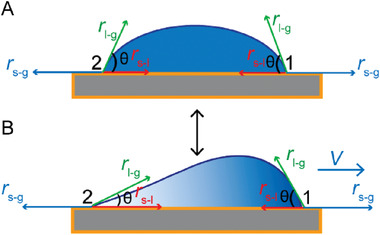
Schematics present the transformation between random motion and directed motion of EDTA nanodroplets.

Upon achieving the directed motion of nanodroplets, we now turn to the question: What determines their sliding direction? When a small macroscopic liquid droplet sits on a smooth flat and isotropic solid surface, gravity is negligible, so that the equilibrium shape is a spherical cap with a contact angle, *θ*.^[^ ^31^ ^]^ In our system, the nanodroplets are self‐shaping all the time under the electron beam, leading to uneven mass distribution. The fact that Si_3_N_4_ membrane possesses charge further exacerbates the displacement of the mass center, so their contact angle at the rear end is influenced more than the front end.^[^ ^32^ ^]^ Accordingly, the simulated charge distribution resulted from the EDTA molecule distribution is also inhomogeneous as shown in Figure S3, Supporting Information. The simulation results suggested that the nanodroplets moved toward the direction of high EDTA concentration, which agrees with the TEM observations. Once the interfacial pressure is strong enough to overcome the resulting hysteresis during the spreading process, nanodroplets move toward the high‐concentration end owing to the driving force produced by the contact angle variation from front and rear end, as shown in **Scheme** **1**. Our results show that these nanodroplets need to experience random motion before starting their directed slide motion. We believe that a strong skewness of mass distribution is required to break the symmetry of droplet shape and induce directed motion. The extent of the skewness is related to the nonuniform distribution of highly negative charged EDTA molecules in the nanodroplets (Zeta potential of the EDTA solution = −16.7 mV). Furthermore, the negatively charged TEM electron beam helps nanodroplets carry on with continuous movements.

**Figure 3:**
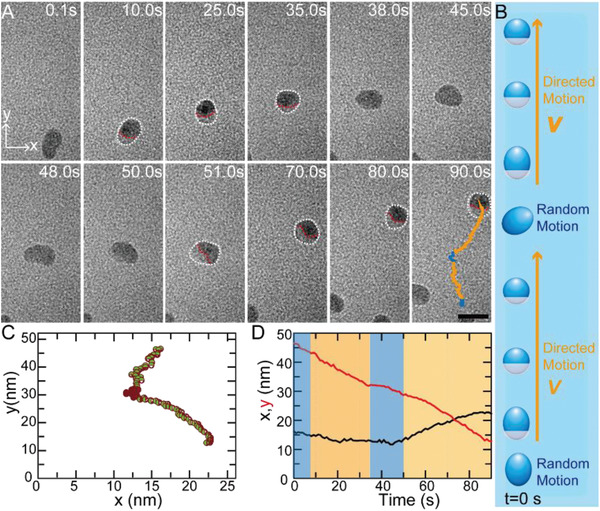
A) In situ TEM images and B) schematic figure show the repeatable switch process from the random motion to the directed motion of EDTA nanodroplets, along with its corresponding C,D) paths within 90 s. The scale bar is 10 nm.

Above results proved the trigger of directed motion of nanodroplet on the hydrophilic surface, yet random motion restores after the mass (as well as charge) distribution is relaxed back to being homogeneous, which can be attributed to the joint force of gravity and the inner flow due to the directed motion. Then it is of importance whether they can restart the directed motion again or not once they are back to the random motion state. In other words, the transformation from random motion to directed motion can happen once or multiple times. Significantly, a long timescale in situ observation of movements of a nanodroplet shows the restart of directed motion (**Figure** **3** and Figure S4, Supporting Information). As shown in Figure 3A, the nanodroplet changed from random motion (from *t* = 0.1 s to *t* = 7 s) to directed motion (from *t* = 7 s to *t* = 35 s) under an electron flux of 150 e (Å^2^·s)^−1^. During the second random motion stage (from *t* = 35 s to *t* = 50 s), the electron flux decreased to 75 e (Å^2^·s)^−1^; still it can restart the directed slide motion (from *t* = 51 s to *t* = 90 s) toward the same direction as before. The total travelling distance is quite appreciable as within two stages it travelled ≈40 nm within 90 s for a droplet only ≈7 nm‐in‐diameter. It means that even if the directed motion at each stage is short, it is still possible to achieve long‐distance movement by combining multiple directed slide motions.

Interestingly, the mean speed of the nanodroplet is accelerated after transforming from random motion to directed motion as shown in Figure S5, Supporting Information, which implied that their energy increased after the periods of random motion. The input energy is considered to be equal to the energy loss when TEM electron beam penetrated the nanodroplets. The mean energy loss, $\left(\frac{dE}{dx}\right)$, for every unit penetration in a sample in TEM is given by the Bethe–Bloch equation, and $\left(\frac{dE}{dx}\right)$ = 3.4 × 10^−11^ J m^−1^ for water.^[^ ^33^ ^]^ Taking the diameter of nanodroplet in Figure 3 which is ≈7 nm, the input energy could be calculated, giving ≈1.5 eV (2.4 × 10^−19^ J), which is much higher than the thermal energy *k*_B_ *T* = 0.026 eV (4.1 × 10^−21^ J; *k*_B_ = Boltzmann constant, *T* = 294 K).^[^ ^23^ ^]^ Therefore, the directed motion overwhelms the random motion. Figure 3 also proved that the directed motion of nanodroplets can be repeated again once they complete the accumulation of energy. Moreover, the time of accumulation energy is proportion to the intensity of electron flux. As the electron flux decreased from 150 to 75 e (Å^2^·s)^−1^, the time increases from ≈7 to ≈15 s. Significantly, after electron flux decreased from 150 to 75 e (Å^2^·s)^−1^, the average speed of random motion and directed motion decreased from 0.88 and 1.04 nm s^−1^ to 0.78 and 0.95 nm s^−1^, respectively, which implied that their motion is highly influenced by the electron flux of TEM. Furthermore, the trajectory curves presented in Figure 3C quantitatively show its travelling distance in *x* and *y* direction, which is ≈6 and ≈35 nm, respectively. Combined with other trajectory curves such as the presented travelling distance direction, it should not be accidental that distance at *y* direction is much longer than *x* direction. Hence, it is possible to achieve continuous driving of the nanodroplets for unidirectional motion on the hydrophilic solid surface. All these results proved that the directed motion of nanodroplets can be restarted from random motion. In other words, this is a reversible transformation process. Therefore, long range movement toward the targeted place could be achieved by triggering multiple directed slide motions, which can be realized through forming the EDTA nonuniform distribution to control the movement direction and providing sufficient input energy to keep their directed motion. According to our results, most of the nanodroplets could continuously carry on the directed motion longer than 30 s and the time of random motion is less than 9 s. On the other hand, the movements of the nanodroplets also present size effect. Nanodroplets smaller than 10 nm behave much better as most of them can support longer time of directed motion while experiencing shorter random motion time. For instance, some nanodroplets could undergo continuous directed motion for ≈120 s. Further study suggested that the liner ratio between the directed motion time and random motion time is larger than 20 (Figure S6, Supporting Information). It means that for most of the nanodroplets, directed motion time accounts for ≈95% on average during their entire movement. We believe that our strategy of in situ preparing nanodroplets and triggering their continuous directed motion can play an important role in many fields such as transporting.

**Figure 4:**
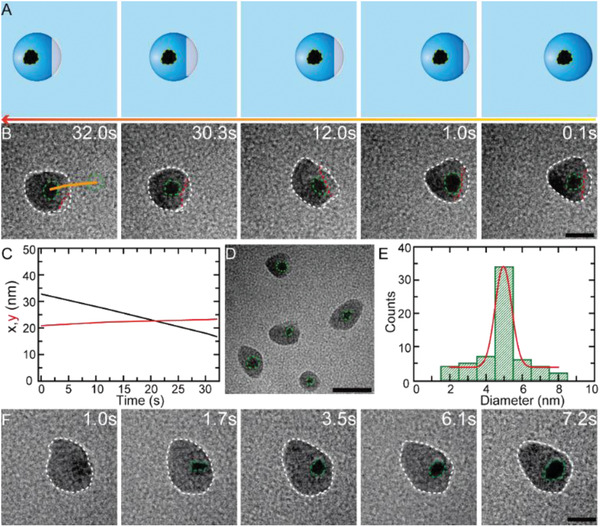
A) Schematic figure and B) in situ TEM images show the directed delivering process of Ag nanoparticle by using nanodroplets, along with its corresponding C) paths during 32 s. D) In situ TEM image of the Ag nanoparticles carried by nanodroplets. E) Histogram of the diameter of the Ag nanoparticles (5 ± 3 nm) in the nanodroplets, measured from ≈60 Ag nanoparticles. F) In situ TEM images present the formation and growth process of an Ag nanoparticle in the nanodroplet. The scale bar is 10 nm.

To demonstrate the nanoreactor and transporting application of these nanodroplets, we used the nanodroplets as microchemical reactors and added AgNO_3_ (0.5 mm) into the nanodroplet solution. Then TEM electron beam was used to in situ reduce the Ag^+^ to Ag^0^ and form Ag nanoparticles. **Figure** **4**B presents the dynamics of transporting process of Ag nanoparticle in a nanodroplet. The trajectory curves presented in Figure 4C quantitatively show the travelling distance of Ag nanoparticle at *x* and *y* direction, which are ≈15 and ≈1.5 nm, respectively. It can be seen that the travel distance in *y* direction can be neglected compared with the distance in *x* direction. It clearly shows that the unidirectional transportation on the hydrophilic isotropic surface has been successfully achieved by using our nanodroplets without gradient driving force. Interestingly, all of these nanodroplets only contain one Ag nanoparticle located at their center, as shown in Figure 4D. These in situ prepared Ag nanoparticles possess narrow size distribution with diameter 5 ± 3 nm (Figure 4E). Our results suggest that the EDTA nanodroplets can not only achieve directed transport of the Ag nanoparticles but also quantitatively control the number of the transported nanoparticles. According to previous reports, when nanoparticles designed for medical purposes entered into cells, a series of interactions leads to their assembly onto the surface of cells, named the “corona” effect.^[^ ^34^ ^]^ A new strategy that deliver droplets including those of precursor solution of nanoparticles not directly delivering nanoparticles into the cells for avoiding “corona” effect can be provided from our work. It is found that the mixed aqueous solution containing ascorbic acid (0.2 mm) and EDTA (2 mm) also could form nanodroplets after being loaded in the liquid cell. Dozens of Ag nanoparticles with size of ≈2 nm were produced in the nanodroplet which were formed through merging ascorbic acid–EDTA nanodroplets with EDTA nanodroplet containing AgNO_3_, as shown in Figure S7, Supporting Information. These nanoparticles also experienced the nucleation and growth process, which were completed within 10.6 s. It implies that these nanodroplets possess very promising applications in many fields such as drug delivery. Furthermore, the formation mechanism of these Ag nanoparticles has been investigated, and the time sequence images of typical growth dynamics of Ag nanoparticles in the nanodroplets are shown in Figure 4F. It shows that the Ag clusters were nucleated first owing to the Ag^+^ ions reduced by TEM electron beam, then gradually merged and grew into larger sized nanoparticles. Eventually, an Ag nanoparticle with diameter of ≈8 nm was formed with a total formation time of 7.2 s.

**Figure 5:**
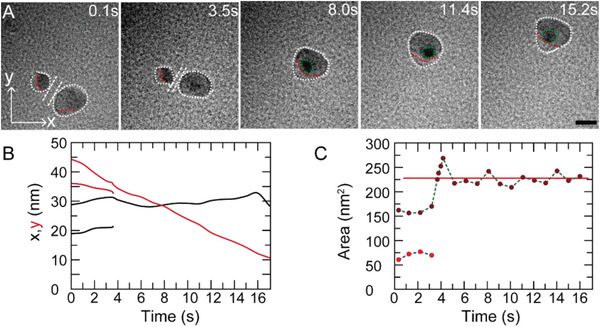
A) In situ TEM images show the coalescence, growth of Ag nanoparticles, transporting process of nanodroplets and their directed motion along with their corresponding B) paths and C) surface area during 16 s. The scale bar is 10 nm.

The observation of a ≈20 nm‐in‐diameter nanodroplet moving on the Si_3_N_4_ surface further confirmed their application in the field of nanoreactor and transporting, as shown in **Figure** **5**A. The directed slide motion of the nanodroplet lasted ≈16 s and the trajectory curves presented in Figure 5B quantitatively show that the displacement of the nanodroplet in *x* and *y* direction is ≈4 and ≈32 nm, respectively. Furthermore, the directed slide motion of the nanodroplet can continue for a longer time (at least 70 s), as shown in Figure S8, Supporting Information. The surface area of the new nanodroplet fluctuated at ≈225 nm^2^ as the change of time (Figure 5C). The results shown in Figure 5 not only confirm the spread‐withdrawal behavior of nanodroplets again, but also indicate that the directed motion is accompanied by the self‐shaping throughout the entire process except for the temporary disturbance due to the coalescence. We found that the synthesis of Ag nanoparticles in the nanodroplets progress simultaneously with the transportation of nanodroplets. Moreover, a nanodroplet can also grow through coalescence and continue the directed slide motion as a bigger droplet on the isotropic hydrophilic solid surface. Evidenced in Figure 5, two nanodroplets approached each other (from *t* = 0.1 s to *t* = 3.5 s), until a tiny liquid bridge connected them (at *t* = 3.6 s) and grew larger, leading to the eventual coalescence (at *t* = 4.0 s). After a short time mixing, they merged and a new nanodroplet formed. It is reported that coalescence of droplets results in the decrease of their surface free energy, so the coalescence process is spontaneous when two droplets are close enough.^[^ ^35^ ^]^ To validate this argument, we calculated the change of surface energies (∆*E*) before and after coalescence according to formula Δ*E* = Δ*Aγ*, Where *γ* is the surface tension (0.0728 N m^−1^) and Δ*A* is the area change of the nanodroplets before and after coalescence (188.1 − 55.7 − 163.7 = −31.3 nm^2^). For the case shown in Figure 5A, Δ*E* ≈ −2.3 × 10^−18^ J, coincides with the change of surface energy for splitting a nanodroplet from a liquid layer, ≈2.4 × 10^−18^ J. According to our results, the detailed coalescence process of nanodroplets is similar with the macro droplets,^[^ ^36^ ^]^ but the formed new nanodroplets would still maintain the moving state after coalescence owing to the high surface free energy; therefore, how to control their moving direction becomes the key factor. Significantly, it seems like that these formed new nanodroplets can keep the same movement direction as the previous bigger one, as the moving direction of the new nanodroplet (*t* = ≈11.1–16.0 s) is almost the same as that of the bigger nanodroplet before coalescence at *t* = ≈0.1–3.0 s. The combination of coalescence without changing the moving direction and directed slide motion on the hydrophilic surface suggests that these nanodroplets can be well‐manipulated and possess tremendous potential applications in many fields such as cleaning wastes, transporting, and collecting nanoscale substances.

## 3 Conclusion

We provide a simple method for generating monodispersed nanodroplets and real‐time conducting their directed motion, and then demonstrate their applications in nanoreactor for controllable in situ synthesis and transportation of Ag nanoparticles by employing in situ liquid cell TEM. The in situ TEM observations showed that the aqueous nanodroplets could exhibit directed motion on the hydrophilic surface. We further found that the transformation from random motion to directed motion due to nonuniform solute distribution is reversible. Furthermore, these nanodroplets can be considered as good nanoreactors for in situ preparing Ag nanoparticles. The diameter and number of theses Ag nanoparticles inside the nanodroplets can be well‐controlled. We also find that the synthesis of Ag nanoparticles in the nanodroplets can be processed with the transportation of nanodroplets at the same time, and the disturbance from nearby nanodroplets will not cease their directed motion. All these results indicate that these nanodroplets can be considered to be an excellent nanoreactor, carrier, and transporter as they can approach their targeted place through triggering multiple directed motions. In a word, we provide a new strategy for generating monodispersed nanodroplets and confirmed their delivery ability by manipulating their unidirectional motion for transporting in situ prepared Ag nanoparticles which nucleated and grew inside the nanodroplets. Also, in situ observed dynamics of generating, splitting, coalescence, and movements of nanodroplets illustrated that they are different from that of macro droplets due to their higher specific surface energy. We believe that our results can also play an important role in many fields such as nanorobotics, micro extraction, micro collecting, micro cleaning, and delivery.

## 4 Experimental Section

##### Chemicals

All the chemicals, including ethylenediaminetetraacetic acid disodium salt (EDTA, ≥99%), sodium hydroxide (NaOH, ≥99%), ascorbic acid (AA, ≥99%) and silver nitrate (AgNO3, ≥99.8%) were purchased from Sigma‐Aldrich company and used without further purification.Deionized (DI) water (Millipore, resistivity >18.2 MΩ) was used throughout the whole experiments.

##### Sample Preparation

A fresh 10 mm EDTA solution for each experiment was prepared by mixing EDTA powder with deionized water. Then, drops of 1 m NaOH aqueous solution were gradually added until EDTA was completely dissolved in water (pH = 12). Next, 200 µL of the 10 mm EDTA aqueous solution was mixed with 800 µL water to form 2 mm EDTA solution (pH = 11.4). A liquid cell was filled with ≈500 nL of this solution and the loading pockets of the liquid cell were sealed with a gasket. Finally, the liquid cell was loaded into a custom TEM holder and inserted into TEM for imaging.

##### Liquid Cell Fabrication

Briefly, the liquid cells were assembled from two chips, each with a 20 nm thick Si_3_N_4_ membrane window (window size: 300 µm × 90 µm) at the center. The top chip contained two pockets for loading the solution, and the bottom chip had ≈100 nm thick spacer to provide separation between the two membranes. After aligning top and bottom chips based on the best overlap of their central windows, the two chips were glued together to form a single liquid cell.

##### Imaging

A JEOL 2010 TEM operated at 200 kV was used for in situ imaging with electron doses ranging from 50 to 100 e (Å^2^·s)^−1^. The movies were acquired at a rate of ten frames per second at 2 × 2 binning (1024 × 1024 pixels). For particle tracking and analyzing, software Fiji Image J was used.

##### Characterization

Zeta potential was measured at ex situ by a Malvern ZetaSizer Nano ZS instrument.

##### Statistical Analysis

For original research, the following information is provided: the diameter of nanodroplets and Ag nanoparticles were obtained from the SEM analysis, accompanied by an average side length of 10 ± 5 and 5 ± 3 nm, respectively. All these data were carried out with Image J (V 1.4) and plotted with OriginPro (V9.0, OriginLab Corp.). The histogram results of the size of nanodroplets and the diameter of the Ag nanoparticles were included in the legends of figure 1 and figure 4, respectively. The EDTA charge distribution inside the droplet was simulated through finite element method by using COMSOL Multiphysics.

## Conflict of Interest

The authors declare no conflict of interest.

## Supporting information

Supporting Information

Supplemental Movie 1

Supplemental Movie 2

Supplemental Movie 3

Supplemental Movie 4
