# Supplementary material for: Generation of Nanodroplet Reactors and Their Applications in In Situ Controllable Synthesis and Transportation of Ag Nanoparticles
Source: Adv Sci (Weinh). 2021 Jan 29;8(6):2002672. doi: 10.1002/advs.202002672 (PMC7967049; doi:10.1002/advs.202002672)
Supplement: Supplementary file 1 — Supporting Information [file ADVS-8-2002672-s002.pdf]

© 2020 Wiley-VCH GmbH

ADVANCED SCIENCE
Open Access

Supporting Information

for *Adv. Sci.,* DOI: 10.1002/advs.202002672

Generation of Nanodroplet Reactors and Their Applications of *in situ* Controllable Synthesis and Transportation of Ag Nanoparticles

Guanhua Lin [a], Haifei Wang [b], Wensheng Lu [b]*

Supporting Information

Generation of Nanodroplet Reactors and Their Applications of *in situ* Controllable Synthesis and Transportation of Ag Nanoparticles

Guanhua Lin [a]*, *Haifei Wang* [b], *Wensheng Lu* [b]

[a] *Institute for Advanced Study, Shenzhen University, Nanhai Avenue 3688, Nanshan District, Shenzhen 518060, Guang Dong Province, China.*

[b] *Beijing National Laboratory for Molecular Sciences, CAS Key Laboratory of Colloid Interface and Chemical Thermaldynamics, Institute of Chemistry, Chinese Academy of Sciences, Beijing 100190, P. R. China*

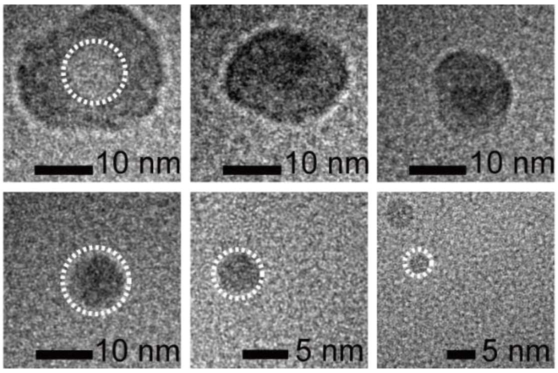

Figure S1. *In situ* TEM images of various sized nanodroplets.

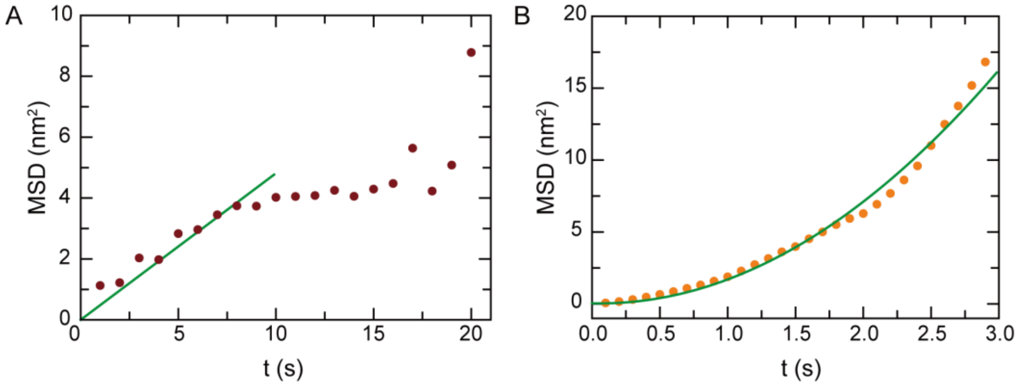

Figure S2. Mean square displacement of droplets in Figure 2. (A) random motion, the diffusion coefficient is about 1.2×10^{-19} m^2/s; (B) directed motion, the velocity of directional motion is about 1.2 nm/s.

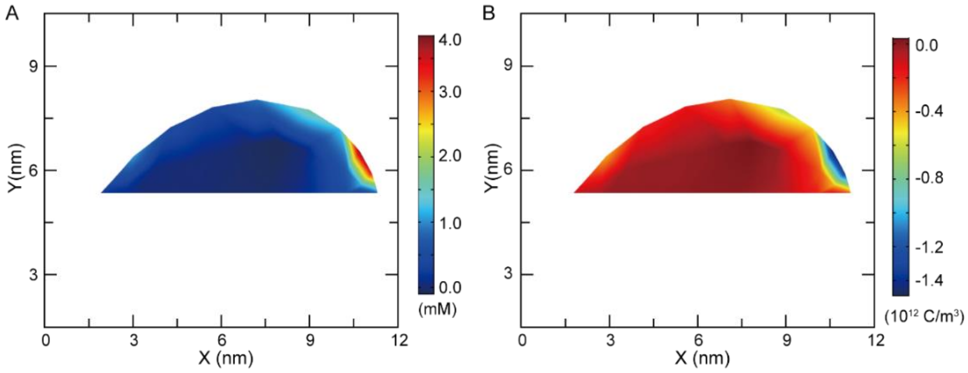

Figure S3. Simulation results for calculating the deformed nanodroplet of (A) EDTA concentration distribution and (B) charge density distribution resulted from the EDTA molecule distribution.

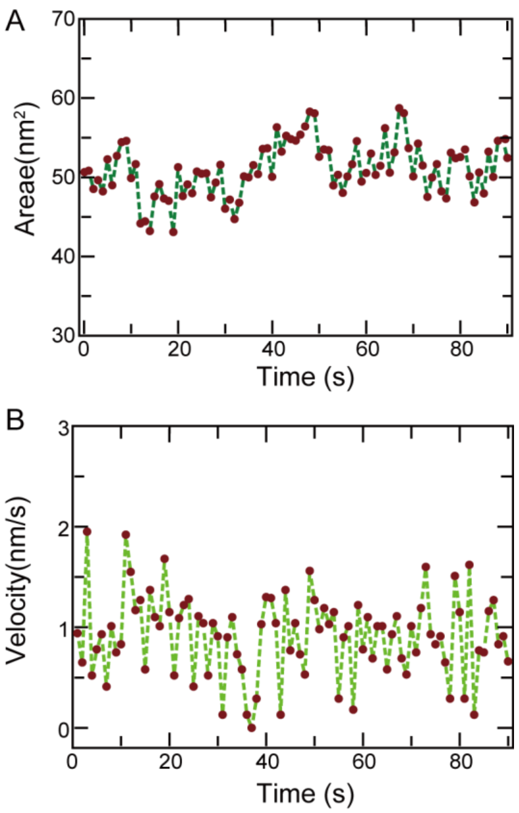

Figure S4. (A) Surface area and (B) velocity change with time analyzed from *in situ* TEM images show the switch process from random shape spontaneous transformation to the direction motion of an EDTA nanodroplets along with its corresponding paths during 90 s.

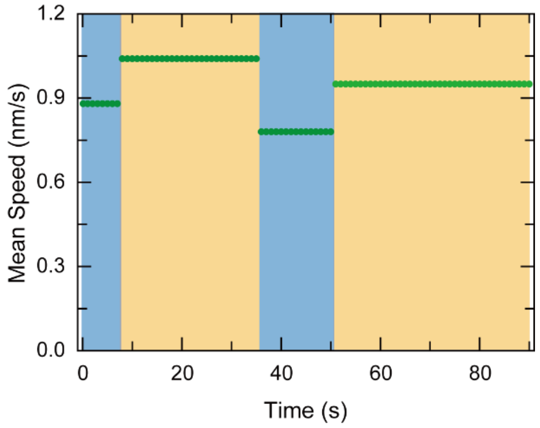

Figure S5. The mean speed of the nanodroplet in Figure 3 at various stages during the repeatable switch process from random transformation to the direction motion in 90 s.

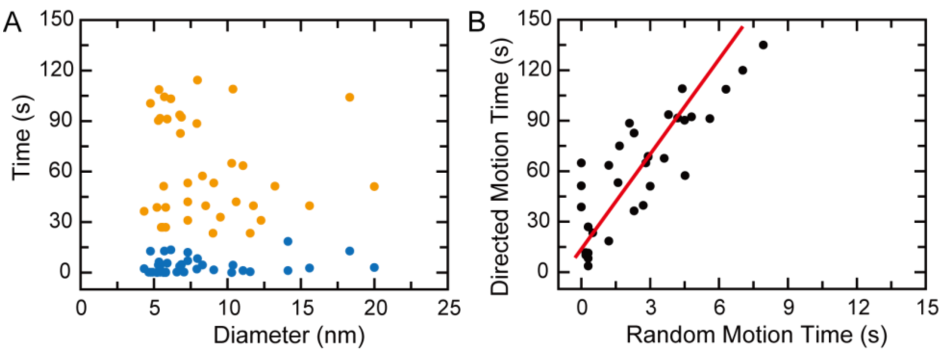

Figure S6. (A) Scattering distribution of the diameter of nanodroplets *VS* the accumulation time of inputting energy (blue) and directed motoion time (organe), (B) and the scattering distribution of the nanodroplets of directed motoion time *VS* random motion time. The data measured from about 40 nanodroplets.

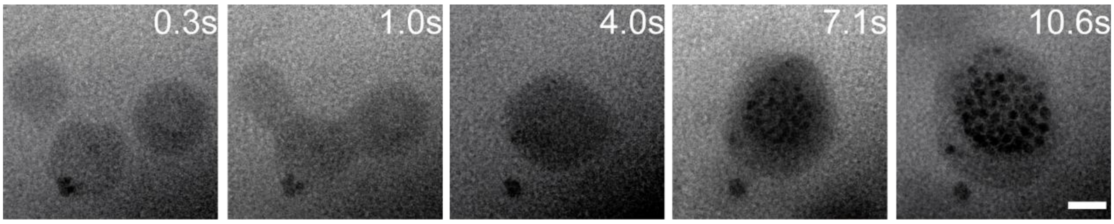

Figure S7. (A) *In situ* TEM images show the dynamical process of Ag nanoparticles growth, which produced by using ascorbic acid (0.2 mM) as the reducing agent. The scale bar is 10 nm.

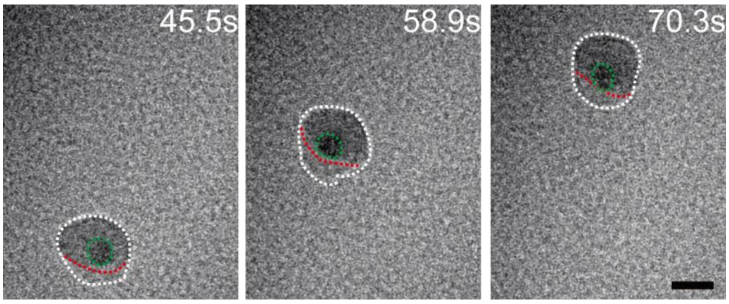

Figure S8. *In situ* TEM images show the transporting process of nanodroplet and the nanoparticle. The scale bar is 10 nm.

Movies

Movie S1. Movie of the generation of three EDTA nanodroplets from liquid layer. Five times faster speed play.

Movie S2. Movie of the random motion of an EDTA nanodroplets. Five times faster speed play.

Movie S3. Movie of the repeatable switch process from random shape spontaneous transformation to the direction motion of an EDTA nanodroplets. Five times faster speed play.

Movie S4. Movie of the unidirectional transporting of Ag nanoparticles by using the directed moved nanodroplets. Five times faster speed play.
